# Thrombosis Related* ABO*,* F5*,* MTHFR,* and* FGG* Gene Polymorphisms in Morbidly Obese Patients

**DOI:** 10.1155/2016/7853424

**Published:** 2016-11-23

**Authors:** Kristina Kupcinskiene, Martyna Murnikovaite, Greta Varkalaite, Simonas Juzenas, Darius Trepenaitis, Ruta Petereit, Almantas Maleckas, Juozas Kupcinskas, Andrius Macas

**Affiliations:** ^1^Department of Anesthesiology, Lithuanian University of Health Sciences, Eiveniu Str. 2, LT-50009 Kaunas, Lithuania; ^2^Institute for Digestive Research, Lithuanian University of Health Sciences, Eiveniu Str. 2, LT-50009 Kaunas, Lithuania; ^3^Department of Gastroenterology, Lithuanian University of Health Sciences, Eiveniu Str. 2, LT-50009 Kaunas, Lithuania; ^4^Department of Surgery, Lithuanian University of Health Sciences, Eiveniu Str. 2, LT-50009 Kaunas, Lithuania

## Abstract

*Objective*. Obesity is a well-known risk factor for thrombotic complications. The aim of the present study was to determine the frequency of thrombosis related* ABO*,* F5*,* MTHFR,* and* FGG* gene polymorphisms in morbidly obese patients and compare them with the group of nonobese individuals.* Methods*. Gene polymorphisms were analyzed in 320 morbidly obese patients (BMI > 40 kg/m^2^) and 303 control individuals (BMI < 30 kg/m^2^) of European descent.* ABO* C>T (rs505922),* F5* C>G (rs6427196),* MTHFR* C>T (rs1801133), and* FGG* C>T (rs6536024) SNPs were genotyped by RT-PCR.* Results*. We observed a tendency for* MTHFR* rs1801133 TT genotype to be linked with morbid obesity when compared to CC genotype; however, the difference did not reach the significant *P* value (OR 1.84, 95% CI 0.83–4.05, *P* = 0.129). Overall, the genotypes and alleles of rs505922, rs6427196, rs1801133, and rs6536024 SNPs had similar distribution between morbidly obese and nonobese control individuals. Distribution of height and weight means among individuals carrying different rs505922, rs6427196, rs1801133, and rs6536024 genotypes did not differ significantly.* Conclusions*. Gene polymorphisms* ABO* C>T (rs505922),* F5* C>G (rs6427196),* MTHFR* C>T (rs1801133), and* FGG* C>T (rs6536024) were not associated with height, weight, or morbid obesity among European subjects.

## 1. Introduction

Obesity has become one of the major health care challenges worldwide. It is linked with multiple medical conditions, significantly affecting longevity and quality of life [1]. Obesity is a well-known risk factor for developing deep venous thrombosis (DVT) [2]. Epidemiological studies show a clear link between body mass index (BMI) and the risk of DVT or related conditions [3, 4]. BMI is one of the major determinants of patients' outcomes in health care emergencies and elective surgery [5]. Development of thrombotic events in obese individuals is also associated with longer hospital admissions and mortality rates [6, 7]. More recently, a number of important research papers have linked the length of legs [8] and height [9] with recurrent venous thrombosis or pulmonary embolism [10]. To date, there are multiple prophylactic antithrombotic regiments; however, risk stratification strategies for prevention of DVT based on anthropometric data need to be improved [11].

Thrombosis and obesity are complex epidemiologically associated diseases, but the mechanism of this association is not yet understood [12]. Development of DVT in obese individuals is thought to result from a complex interaction of host and environmental factors [13]. The pathogenesis of DVT has been linked with chronic low grade inflammation, heritability, diet, physical activity, and other potential risk factors [13]. Recent advances in molecular genotyping techniques outlined the importance of genetic factors for development of thrombosis [14]. It is estimated that more than 60% of the variation in susceptibility to common thrombosis might be attributable to genetic factors [15].

To date, there are several genome wide association studies (GWAS) that have linked various genetic factors with the risk of developing thrombotic complications. Trégouët et al. conducted a GWAS by analyzing approximately 317,000 single nucleotide polymorphisms (SNPs) in 453 venous thromboembolism (VTE) cases and 1327 controls and found that three SNPs located in the* F5* and* ABO* blood group genes were associated with VTE at a genome wide significant level [16]. Another comprehensive genome wide association analysis, testing 336,469 SNPs in 13,974 healthy Caucasian women, confirmed the association of* MTHFR* (rs1801133) and* CBS* (rs6586282) SNPs with homocysteine levels that have been linked with thrombotic events [17]. A large GWAS including nearly 45,000 individuals reported key genetic associations in* F5*,* ABO*, and* FGG* loci for VTE [18]. The same loci at* F5*,* ABO*, and* FGG* have been linked with VTE in another study including 1,542 cases and 1,110 controls [19]. The results of these GWAS studies have been replicated in smaller case-control studies [20]; however, the frequencies of these genetic variations have not been assessed in morbidly obese patients. Furthermore, they have not been previously analyzed in relation to height and weight.

Souto et al. have shown that BMI and thrombosis are genetically linked [12]. They showed that both venous and arterial thromboembolic disease and BMI had a significant genetic correlation. A Danish study observed a strong observational association between obesity and DVT with or without pulmonary embolism (PE), supported by a direct genetic association between the obesity-specific genetic loci and DVT with PE [21]. Studies discussed above clearly imply that obesity might likely be causally associated with DVT.

The aim of our present study was to determine the frequencies of thrombosis related* ABO*,* F5*,* MTHFR,* and* FGG* gene polymorphisms in morbidly obese patients and compare them with the group of nonobese individuals. Frequencies of* ABO* C>T (rs505922),* F5* C>G (rs6427196),* MTHFR* C>T (rs1801133), and* FGG* C>T (rs6536024) gene polymorphisms have not been previously evaluated in morbidly obese patients. We also aimed to evaluate whether the genotypes of above-mentioned gene polymorphism are linked with height or weight of study individuals. Here, in this study we performed* ABO*,* F5*,* MTHFR,* and* FGG* SNP genotyping analysis in 320 morbidly obese patients (BMI > 40 kg/m^2^) and 303 control nonobese individuals (BMI < 30 kg/m^2^) of European descent.

## 2. Materials and Methods

### 2.1. Study Population

The group of morbidly obese subjects consisted of patients referred for elective bariatric surgery with a BMI >40 kg/m^2^. Control subjects were healthy individuals with BMI <30 kg/m^2^, who came from our previous genotyping studies [22, 23]. Morbidly obese patients and controls were recruited during the years 2011–2015 in the Departments of Surgery and Gastroenterology, Lithuanian University of Health Sciences (Kaunas, Lithuania). The inclusion criteria for control group were no previous history of malignancy, VTE, and BMI <30 kg/m^2^. In total, 623 individuals (303 controls and 320 morbidly obese patients) were included in the genotyping study. All patients were of European ethnicity. The study was approved by Kaunas Regional Ethics Committee (Protocol number BE-2-10). All patients have signed an informed consent form to participate in this genotyping case-control study.

### 2.2. DNA Extraction and Genotyping

Genomic DNA of study participants was obtained from peripheral blood mononuclear cells using phenol-chloroform extraction method as described previously [22]. DNA aliquots were stored at −20°C until RT-PCR analysis. SNPs of* ABO* T>C (rs505922),* F5* C>G (rs6427196),* MTHFR* G>A (rs1801133), and* FGG* T>C (rs6536024) were genotyped using custom TaqMan® assays with a 7500™ real-time cycler according to manufacturer's instructions (Life Technologies, CA, USA). Thermal cycling conditions for polymerase chain reaction (PCR) were, first, denaturing at 95°C for 10 min, followed by 40 cycles of 95.5°C for 15 s and 60°C for 1 min. Alleles and genotypes of analyzed SNPs were determined with SDS 2.0.5 software compatible.

### 2.3. Genotyping Quality Control

5% of samples for each of the four SNPs were selected for repetitive analysis. Replication experiments revealed a 100% concordance rate of genotypes and alleles with the initial genotyping results.

### 2.4. Statistical Analysis

All study participants were stratified into two groups: 320 morbidly obese patients (BMI > 40 kg/m^2^) and 303 control nonobese individuals (BMI < 30 kg/m^2^). Age, height, weight, and BMI are presented as mean and standard deviations and was compared using unpaired Student's *t*-test. Categorical data (gender, distribution of genotypes or alleles) are presented as frequencies; comparisons were performed using the Chi-square test. Quality assessments and statistical analysis of genotyping data were carried out using free PLINK software (version 1.07) for genetic analysis [24]. Association of morbid obesity with gene polymorphisms was calculated using logistic regression analysis with adjustment for age and gender and presented as adjusted odds ratios (aOR) with 95% confidence intervals (CI). The relative risks for SNP genotypes and alleles were studied using recessive and dominant models that led to a comparison between wild type + heterozygous versus homozygous and wild type versus heterozygous + homozygous, respectively. Due to multiple association calculations we introduced an adjusted significance threshold for multiple comparisons *α* = 0.0125 (0.05/4). One way ANOVA or unpaired *t*-test was used to compare height and weight differences between different genotypes of each SNP.

## 3. Results

### 3.1. Characteristics of the Subjects

Characteristics of control and morbidly obese patient groups are presented in Table 1. In total 623 individuals participated in the study (320 morbidly obese and 303 control subjects). Individuals in the control group were significantly older than morbid obesity group subjects, 61.5 and 42.6 years, respectively (*P* < 0.001). Males accounted for 60.9% in a group of patients with BMI >40 kg/m^2^, while in the control group they constituted 42.6% (*P* < 0.001). Evidently, mean BMI in morbidly obese group was significantly higher (46.0 kg/m^2^) than compared to control group (25.1 kg/m^2^, *P* < 0.001). Since proportion of males and females as well as age were significantly different between the two groups, gender and age were included as confounding factors in further logistic regression analysis of genotyping results.

### 3.2. Hardy-Weinberg Equilibrium

All four* ABO* T>C (rs505922),* F5* C>G (rs6427196),* MTHFR* G>A (rs1801133), and* FGG* T>C (rs6536024) were tested for Hardy-Weinberg equilibrium. The results of the analysis are presented in Table 2. The conditions of Hardy-Weinberg equilibrium were fulfilled for all of investigated SNPs as the frequencies of observed and expected genotype and allele frequencies did not differ: rs505922, *P* = 0.241; rs6427196, *P* = 1; rs1801133, *P* = 0.235; rs6536024, *P* = 0.418 (Table 2).

### 3.3. Frequencies of rs505922, rs6427196, rs1801133, and rs6536024 Genotypes and Allele

Genotype and allele distributions for* ABO* T>C (rs505922),* F5* C>G (rs6427196),* MTHFR* G>A (rs1801133), and* FGG* T>C (rs6536024) in morbid obesity and control groups are presented in Table 3. All individuals were successfully genotyped for rs505922, rs6427196, and rs6536024 loci, while one individual in morbid obesity group failed genotyping for rs1801133. Overall, the genotypes and alleles of rs505922, rs6427196, rs1801133, and rs6536024 SNPs had similar distribution between morbidly obese and nonobese control individuals. We observed a tendency for* MTHFR* rs1801133 TT genotype to be linked with morbid obesity when compared to CC genotype; however, the difference did not reach the significant *P* value (odds ratio (OR), 1.84, 95% confidence interval (CI) 0.83–4.05, and *P* = 0.129, Table 3). Similar results were obtained in a recessive model for* MTHFR* SNP (TT versus CT + CC), but association remained beyond statistical significance (OR 1.81, 95% CI 0.83–3.90, and *P* = 0.131, Table 3).

### 3.4. Distribution of Height and Weight Means for rs1801133, rs6427196, rs505922, and rs6536024 Genotypes

**Figure 1:**
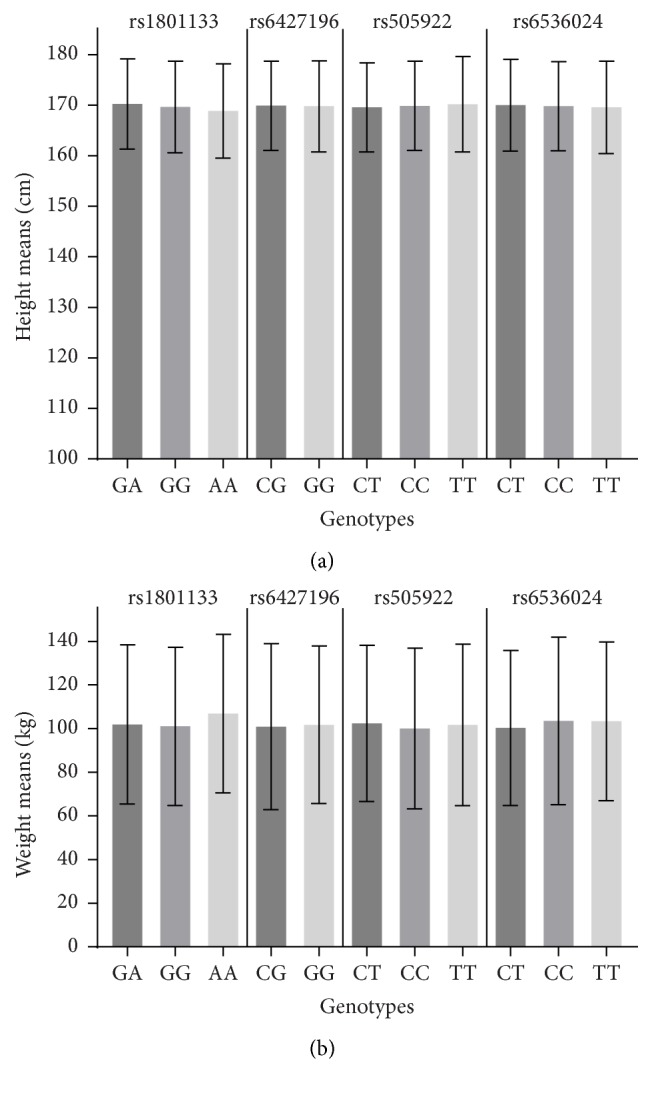
(a) Mean height by SNP (rs505922, rs6427196, rs1801133, and rs6536024) genotypes; (b) mean weight by SNP (rs505922, rs6427196, rs1801133, and rs6536024) genotypes. Error bars indicate the standard deviation in (a) cm and (b) kg in each group. In rs6427196 analysis only one subject was identified as having genotype CC and, therefore, was excluded from analysis.

Distribution of height and weight means for* ABO* T>C (rs505922),* F5* C>G (rs6427196),* MTHFR* G>A (rs1801133), and* FGG* T>C (rs6536024) genotypes for all subjects included in the study is presented in Figure 1. Additionally, one way ANOVA analysis was performed to compare height and weight means distribution between single SNP genotypes separately (Table 4). Rs6427196 analysis showed only one subject which was recognized as genotype CC; therefore, this group was excluded from analysis; height and weight means were compared only for CG and GG genotypes using *t*-test. The results show that height and weight means were similar when compared between different SNPs genotypes (Table 4; Figure 1).

## 4. Discussion

In our study we analyzed* ABO* C>T (rs505922),* F5* C>G (rs6427196),* MTHFR* C>T (rs1801133), and* FGG* C>T (rs6536024) gene polymorphisms in a case-control study including 320 morbidly obese subjects and 303 controls of European descent. These genetic polymorphisms have been associated with the risk of DVT and PE; however, they have not been previously investigated in a group of patients with extreme overweight who are more prone to thrombotic complications. The results of our study show that gene polymorphisms of* ABO* (rs505922),* F5* (rs6427196),* MTHFR* (rs1801133), and* FGG* (rs6536024) are not associated with the presence of morbid obesity among European subjects. We also did not determine differences in height or weight of individuals carrying different rs505922, rs6427196, rs1801133, or rs6536024 genotypes.

Multiple epidemiological and clinical studies showed a clear link between obesity and higher risk of thrombotic complications [2, 4]. The exact mechanisms of DVT development are still poorly understood [13]. Since genetic polymorphisms have been shown to have profound effect on the risk of thrombotic events [25], we expected that* ABO*,* F5*,* MTHFR,* and* FGG* genetic variations might be more prevalent in obese individuals who have higher risk of DVT than nonobese individuals. To date, 17 genes have been robustly demonstrated to harbor genetic variations associated with VT risk: ABO, F2, F5, F9, F11, FGG, GP6, KNG1, PROC, PROCR, PROS1, SERPINC1, SLC44A2, STXBP5, THBD, TSPAN15, and VWF [25]. Furthermore, genetic variations have been showing significant plasma levels of coagulation factors VII (FVII), VIII (FVIII), and von Willebrand factor (vWF) and thus mediate the risk of hemorrhage and thrombosis [26].

Previous studies have uncovered that* ABO* genetic variations are important risk factors for VTE [19, 27, 28]. A GWAS study identified* ABO* rs505922 as a risk factor for venous thrombosis [19]. Additionally, International Stroke Genetic Consortium revealed that SNP rs505922 was nominally associated with ischemic stroke (OR = 0.94, *P* = 0.023) [29]. Our genotyping analysis showed that T and C alleles of rs505922 and corresponding genotypes are distributed equally between the obese and control groups. The results of allele and genotype frequencies in our study are also comparable to the results obtained in previous studies with T allele ranging from 59 to 62% [30]. Interestingly, a recent meta-analysis provided some evidence that* ABO* rs505922 SNP C allele is a risk factor for cancer susceptibility, specifically for pancreatic cancer [30].

F5 is a protein of the coagulation system, which is not enzymatically active but functions as a cofactor [13]. Deficiency of F5 increases the risk of bleeding, while some mutations predispose to thrombosis [31]. Genetic variations in* F5* region have been clearly linked with the risk of thrombosis with Leiden mutation rs6025 being the most well-known [25]. In order to identify additional novel genetic determinants of VTE, Tang et al. conducted a 2-stage GWAS among individuals of European ancestry [18]. This study showed two signals at the* F5* region with an intronic variant rs2420370 and a coding variant rs6427196 in the 3′ untranslated region of* F5*. Within our study genotypes and alleles of rs6427196 were distributed equally between cases and controls.

MTHFR is the rate-limiting enzyme in the methyl cycle which is encoded by the* MTHFR* gene [32].* MTHFR* gene polymorphism has been extensively studied in relation to different medical conditions. To date, there are reports that have linked rs1801133 polymorphism within* MTHFR* gene with arterial hypertension [33], cancer [34], diabetes [35], and many other diseases; however, the results of replication studies vary in between [36]. The homozygous mutated subjects for rs1801133 have higher homocysteine levels and hyperhomocysteinemia is an emerging risk factor for various thrombotic diseases [37]. A large number of studies has clearly shown that* MTHFR* gene polymorphism rs1801133 is a risk factor for thrombotic events [25]. In our study we observed a tendency for* MTHFR* rs1801133 TT genotype to be linked with morbid obesity when compared to CC genotype; however, the difference did not reach required significance. Similar results were obtained in a recessive model for* MTHFR* SNP (TT versus CT + CC), but association remained beyond statistical significance.

Fibrinogen gamma* (FGG)* gene belongs to the fibrinolysis cascade [25]. The genotypes of FGG gene have provided robust positive findings with the risk of VTE [25]. The T allele of the rs2066865 polymorphism in the year 2005 was found to reduce gamma fibrinogen plasma levels and to increase thrombosis risk by a factor of ~1.50 [38]. A more recent large GWAS study confirmed the importance of another* FGG* locus rs6536024 for VTE development [18]. Within our study we observed equal distribution of rs6536024 alleles in control and morbidly obese patient groups. The frequencies of C and T alleles for rs6536024 in our study correspond to previously published data on Caucasian subjects [18].

Several studies have suggested that height or length of legs of individuals is linked with increased risk of thrombotic events [8–10]. Therefore, we performed additional analysis in order to compare means of height and weight according to individual genotype of each of the four SNPs analyzed. In our study the height and weight means between separate* ABO* (rs505922),* F5* (rs6427196),* MTHFR* (rs1801133), and* FGG* (rs6536024) gene polymorphisms did not differ. Interestingly, combination of obesity and tall stature was associated with VTE in Norwegian study [39]. These findings might be due to greater venous surface area, a larger number of venous valves, or greater hydrostatic pressure with longer legs [8]; however, the role of genetic factors within this context remains to be established.

To our best knowledge, this is the first study which investigated the frequencies of rs505922, rs6427196, rs1801133, and rs6536024 in morbidly obese subjects. Nevertheless, we admit that there are several important limitations related to the design of our study. There were gender and age distribution differences between obese and control groups; however, when performing statistical analysis we included gender and age as covariates, thus reducing the potential influence of these factors for the results. We also included only four gene polymorphisms in our study, while other genetic variations might be more important in the pathogenesis of thrombosis in obese individuals. Further large scale studies including obese patients who have the history of thrombotic complications would be extremely valuable. We could only speculate that certain genetic polymorphisms might be included in multivariate risk models for predicting thrombosis in morbidly patients in the future.

## 5. Conclusions

Our study showed that gene polymorphisms* ABO* C>T (rs505922),* F5* C>G (rs6427196),* MTHFR* C>T (rs1801133), and* FGG* C>T (rs6536024) were not associated with morbid obesity, height, or weight in European subjects.

## Figures and Tables

**Table 1:** Characteristics of study groups.

	Morbid obesity group (*n* = 320)	Control group (*n* = 303)	*P* value
Gender (*n*, %)			
Males	195 (60.9)	129 (42.6)	<0.001
Females	125 (39.1)	174 (57.4)	
Age (years)			
Mean ± SD	42.6 ± 11.2	61.5 ± 8.2	<0.001
BMI (kg/m^2^)			
Mean ± SD	46.0 ± 4.2	25.1 ± 2.7	<0.001

BMI, body mass index.

**Table 2:** Analysis of Hardy-Weinberg equilibrium.

SNP	Allele frequencies		Genotype distribution	Determined frequency of heterozygous allele	Expected frequency of heterozygous allele	*P* value
rs1801133	*T* (0.281)	*C* (0.719)	43/264/315	0.424	0.404	0.235
rs6427196	*C* (0.052)	*G* (0.948)	1/63/559	0.101	0.099	1
rs6536024	*C* (0.343)	*T* (0.657)	20/319/184	0.512	0.495	0.418
rs505922	*C* (0.396)	*T* (0.604)	90/313/220	0.502	0.478	0.241

SNP, single nucleotide polymorphism.

**Table 3:** Genotype and allele frequencies of *ABO* C>T (rs505922), *F5* C>G (rs6427196), *MTHFR* C>T (rs1801133), and *FGG* C>T (rs6536024) SNPs in morbidly obese and nonobese control individuals.

Alleles/genotypes	Morbid obesity group (*n* = 320)	Control group (*n* = 303)	aOR	95% CI	*P*
	*n*, %	*n*, %			
rs505922 *ABO *C>T					
T	380 (59.4)	373 (61.6)			
C	260 (40.6)	233 (38.4)	1.10	(0.877–1.38)	0.406
TT	107 (35.3)	113 (37.9)	—		
TC	166 (48.2)	147 (47.3)	1.26	(0.834–1.90)	0.273
CC	47 (16.5)	43 (14.7)	1.06	(0.593–1.91)	0.836
CC + TC versus TT			1.21	(0.819–1.79)	0.337
CC versus TC + TT			0.930	(0.546–1.58)	0.788
rs6427196 *F5* C>G					
G	610 (95.3)	571 (94.2)			
C	30 (4.7)	35 (5.8)	0.805	(0.488–1.32)	0.395
GG	291 (90.8)	268 (88.7)	—		
GC	28 (9.0)	35 (10.9)	0.605	(0.325–1.13)	0.112
CC	1 (0.2)	0 (0)	1.82 × 10^8^	(0–inf)	0.999
CC + GC versus GG			0.613	(0.330–1.14)	0.120
CC versus GC + GG			1.95 × 10^8^	(0–inf)	0.999
rs1801133 *MTHFR *C>T					
C	447 (70.1)	447 (73.8)			
T	191 (29.9)	159 (26.2)	1.201	(0.938–1.54)	0.147
CC	156 (49)	159 (54.5)	—		
CT	135 (42)	129 (38.7)	1.043	(0.706–1.54)	0.832
TT	28 (8.9)	15 (6.9)	1.842	(0.837–4.05)	0.129
TT + TC versus CC			1.127	(0.775–1.64)	0.532
TT versus CT + CC			1.808	(0.838–3.90)	0.131
rs6536024 *FGG* C>T					
T	355 (55.5)	532 (66)			
C	285 (44.5)	274 (34)	0.972	(0.777–1.22)	0.804
TT	100 (30.8)	184 (43.6)	—		
TC	155 (49.4)	164 (44.9)	0.796	(0.516–1.23)	0.303
CC	65 (19.8)	55 (11.6)	1.14	(0.660–1.97)	0.638
CC + TC versus TT			0.881	(0.585–1.33)	0.545
CC versus TC + TT			1.31	(0.817–2.11)	0.260

aOR, adjusted odds ratio by sex and age; CI, confidence interval.

**Table 4:** Mean height and weight by SNPs genotypes (rs1801133, rs6427196, rs505922, and rs6536024).

SNPs	Means ± SD by genotype	*P*
rs505922 *ABO *C>T		
Height (CT/CC/TT)	169.92 ± 9.29/170.67 ± 9.36/171.75 ± 9.05	0.755^1^
Weight (CT/CC/TT)	132.35 ± 24.97/132.95 ± 23.86/135.29 ± 24.67	0.866^1^
rs6427196^3^ *F5* C>G		
Height (CG/GG)	170.04 ± 8.52/170.63 ± 9.22	0.937^2^
Weight (CG/GG)	137.08 ± 30.27/132.95 ± 24.02	0.853^2^
rs1801133 *MTHFR *C>T		
Height (GA/GG/AA)	171.18 ± 9.14/170.41 ± 9.08/169.60 ± 10.63	0.580^1^
Weight (GA/GG/AA)	133.98 ± 26.28/133.77 ± 23.11/129.16 ± 26.35	0.633^1^
rs6536024 *FGG* C>T		
Height (CT/CC/TT)	170.83 ± 9.38/171.35 ± 9.11/169.93 ± 9.11	0.870^1^
Weight (CT/CC/TT)	133.32 ± 23.72/135.53 ± 25.10/132.33 ± 26.04	0.575^1^

^1^One way ANOVA *P* value; ^2^unpaired *t*-test *P* value; ^3^In rs6427196 analysis only one subject was identified as having genotype CC and, therefore, was excluded from analysis.
